# Application of machine learning algorithms to predict the thyroid disease risk: an experimental comparative study

**DOI:** 10.7717/peerj-cs.898

**Published:** 2022-03-03

**Authors:** Saima Sharleen Islam, Md. Samiul Haque, M. Saef Ullah Miah, Talha Bin Sarwar, Ramdhan Nugraha

**Affiliations:** 1 Department of Computer Science, Faculty of Science and Technology, American International University - Bangladesh (AIUB), Dhaka, Bangladesh; 2 Faculty of Computing, College of Computing and Applied Sciences, Universiti Malaysia Pahang, Pekan, Pahang, Malaysia; 3 Faculty of Electrical Engineering, Telkom University, Bandung, Indonesia

**Keywords:** Thyroid Disease, Thyroid Risk Prediction, Machine Learning Algorithm, Machine Learning Classifier, Sick-euthyroid

## Abstract

Thyroid disease is the general concept for a medical problem that prevents one’s thyroid from producing enough hormones. Thyroid disease can affect everyone—men, women, children, adolescents, and the elderly. Thyroid disorders are detected by blood tests, which are notoriously difficult to interpret due to the enormous amount of data necessary to forecast results. For this reason, this study compares eleven machine learning algorithms to determine which one produces the best accuracy for predicting thyroid risk accurately. This study utilizes the Sick-euthyroid dataset, acquired from the University of California, Irvine’s machine learning repository, for this purpose. Since the target variable classes in this dataset are mostly one, the accuracy score does not accurately indicate the prediction outcome. Thus, the evaluation metric contains accuracy and recall ratings. Additionally, the F1-score produces a single value that balances the precision and recall when an uneven distribution class exists. Finally, the F1-score is utilized to evaluate the performance of the employed machine learning algorithms as it is one of the most effective output measurements for unbalanced classification problems. The experiment shows that the ANN Classifier with an F1-score of 0.957 outperforms the other nine algorithms in terms of accuracy.

## Introduction

Thyroid illnesses and disorders are widespread hormonal problems that affect the majority of the world’s population. Thyroid illness and disorders include thyroiditis and thyroid malignancy. The thyroid gland is one of the most conspicuous pure endocrine glands, located at the front of the neck and encircling the trachea (Beynon & Pinneri, 2016). It has the appearance of a butterfly, with two long wings extending to either side of the human neck, and it is responsible for hormone regulation in our bodies. Thyroid hormones are involved in the digestion of fat, protein, and carbohydrates, as well as the regulation of blood pressure, body temperature, and heart rate. On the other side, a shift in secretion can result in a variety of physiological and psychological problems, including brain fog, peripheral neuropathy, goiter, and depression. The two most prevalent thyroid problems are hypothyroidism and hyperthyroidism (Taylor et al., 2018). When the thyroid gland produces excessive thyroid hormone, hyperthyroidism develops, whereas hypothyroidism occurs when the thyroid gland does not enlarge adequately.

T4 (tetraiodothyronine or thyroxine) and T3 (triiodothyronine) are thyroid hormones that help regulate metabolism. Thyrotropin is responsible for the regulation of these two thyroid hormones (TSH). All kinds of hyperthyroidism are caused by an excess of these hormones. Other illnesses, such as serious diseases, can be attributed to excessive hormone production. Toxic adenomas can develop as a result of thyroid nodules leaking thyroid hormones and disrupting the body’s chemical equilibrium. Thyroid inflammation causes the thyroid gland to secrete excessive hormones, resulting in subacute thyroiditis, which normally lasts a few weeks but can last months. Additionally, hyperthyroidism can be caused by problems with the pituitary gland or malignant tumors. Certain conditions, such as thyroid cancer, are inherited from our parents. For example, in two out of every ten instances of medullary thyroid cancer, the tumor is caused by an inherited faulty gene (Pichardo, 2021). Additionally, if our diet is deficient in certain chemical components, we may be at a higher risk of acquiring certain forms of thyroid cancer. These two hormones are produced by the thyroid and are controlled by thyrotropin (TSH). According to the American Cancer Society’s most current forecasts, 44,280 new instances of thyroid cancer (12,150 men and 32,130 women) have occurred in the United States since 2021, and 1,050 men and 1,150 women have died of thyroid cancer. Between 2009 and 2018, the thyroid cancer fatality rate increased dramatically (0.6 percent per year) (Akbas et al., 2021).

Thyroid diseases are difficult to detect in test results, and only trained professionals are capable of doing so Eggertsen et al. (1988). Thyroid diseases are diagnosed in two ways: through blood testing and by symptoms, signs, and physical (Nguyen et al., 2015). However, each research is difficult to understand due to the massive amount of data needed to anticipate results. Alqurashi & Wang (2019) used different clustering algorithms to create an adaptive clustering ensemble model to predict thyroid illness. The adaptive clustering strategy used the K-means algorithm to generate and transform initial clusters into binary representations in order to forecast final clusters. Li et al. (2020) acquired 93.30 percent accuracy in thyroid illness utilizing the AR-ANN technique. They used association rule mining methods to assess the datasets. They found an AUC of 0.9458 using the ROC curve. They then compared their AR-ANN model’s accuracy score to that of other models and observed that it beat Naive Bayes, KNN, and SMO classifiers.

This study examines multiple machine learning algorithms, including CatBoost, Extra-Trees, ANN, LightGBM, SVC, KNN, Random Forest, XGBoost, Decision Tree, and GaussianNB, in order to enhance thyroid prediction accuracy and thereby identify thyroid problems. Thus, if abnormal thyroid hormone levels are recognized early enough, patients may be prescribed the appropriate medicine and therapy. The machine learning algorithms are trained on the thyroid illness dataset from the University of California, Irvine. To summarize, the major contribution is as follows:

• To estimate the likelihood of a better outcome when more data is used, as increasing thyroid prediction accuracy will enhance thyroid problem identification.
• To enhance the model’s performance, a variety of pre-processing techniques like identifying and handling the missing values, encoding the categorical data etc. are applied.
• To evaluate the effectiveness of the employed machine learning algorithms, the accuracy, precision, recall, and F1-scores are examined.

The remainder of this paper is structured in the following way. The related study examines the existing studies regarding thyroid disease, the datasets that accompany this study, and an extensive overview of the employed algorithms. Methodology examines this work’s dataset pre-processing, feature selection techniques, and performance metrics in detail. Additionally, it discusses the implementation of the machine learning models. The Experimental Results and Discussions sections highlight the outcomes from analyzing the thyroid disorder dataset using several classification methods. The performance of several algorithms is then compared. The Conclusion serves as a summary of the entire work.

## Related Study

The thyroid gland is one of the body’s most visible endocrine glands. Its size is determined by the individual’s age, gender, and physiological states, such as pregnancy or lactation (Kumar et al., 2020). It is divided into two lobes (right and left) by an isthmus (a band of tissue). It is imperceptible in everyday life yet can be detected when swallowing. The thyroid hormones T4 and T3 are needed for normal thyroid function. These hormones have a direct effect on the body’s metabolic rate. It contributes to the stimulation of glucose, fatty acid, and other molecule consumption. Additionally, it enhances oxygen consumption in the majority of the body’s cells by assisting in the processing of uncoupling proteins, which contributes to an improvement in the rate of cellular respiration (Leitch, Bassett & Williams, 2020).

Thyroid conditions are difficult to detect in test results (Paczkowska et al., 2020), and only trained professionals can do so. However, reading such extensive reports and predicting future results is difficult. Assume a machine learning model can detect thyroid disease in a patient. The thyroid disease can then be easily identified based on the symptoms in the patient’s history. Currently, models are evaluated using accuracy metrics on a validation dataset that is accessible. In addition to such metrics, inspecting individual forecasts and their descriptions is a realistic approach. In this case, particularly for large databases, it is critical to assist users by recommending which instances to inspect. The thyroid disease dataset was the common dataset on which previous thyroid disease research was published. This section contains a description of previous thyroid disease research as well as classification algorithms that were developed using the Sick-euthyroid dataset (Dua & Graff, 2017).

Numerous studies have been performed to date on various thyroid datasets (Razia, Kumar & Rao, 2020). Currently, the UCI machine learning repository contains ten datasets. Different studies have been conducted based on these ten datasets. In 2006, Polat, Şahan & Güneş (2007) tested the artificial immune-recognition system (AIRS) for thyroid illness detection and discovered that AIRS was 81% accurate. Akbaş et al. (2013) used the thyroid cancer train dataset to train BayesNet, NaiveBayes, SMO, Ibk, and random forest classifiers. When classification accuracy and receiver operating characteristic (ROC) plots are compared, the random forest (99%) method outperforms other methods in the diagnosis of thyroid cancer. When past researches are included, the accuracy of the classification method in this dataset has been observed to be very near to 100%. Making forecasts with such great accuracy complicates thyroid cancer research. Ahmad et al. (2017) used data mining categorization to address thyroid-related disorders. They conducted a three-stage analysis of the dataset and discovered 99.1% classification accuracy using KNN.

Parikh et al. (2015) discuss the two prediction models they developed to solve their multiclass classification problem. They used artificial neural networks and support vector machines and achieved an accuracy of 97.17 percent with the ANN. Ozyilmaz & Yildirim (2002) pioneered the use of an artificial neural network to diagnose thyroid disease, and they examined many neural network models, including backpropagation (MLP with backpropagation), radial basis function (RBF), and adaptive conic section function (CSFNN). Ioniţă & Ioniţă (2016) explored hybrid medical datasets and described several applications of Naive Bayes, Decision trees, MLP, and RBF networks. All classifiers categorize and produce distinct outcomes for classification accuracy, however a decision tree achieved 97.35% accuracy. Talasila et al. (2020) demonstrated and tested various methods, concluding that LightGBM produced more accurate predictions than other available methodologies. However, accuracy for EF using LightGBM remained low.

Keleş & Keleş (2008) introduced ESTDD, an expert method for diagnosing thyroid disease. However, it is only suitable for small data sets and cannot be used in operation due to the technology’s limitations. Bastias et al. (2011) set out to develop a machine learning classifier capable of diagnosing the health concerns and exploring the suggested classifier’s capabilities. The proposed classifier considerably aided in the process of thyroid gland illness identification.

Azar, El-Said & Hassanien (2013) assessed thyroid illness using compression hard and fuzzy clustering techniques and identified the optimal number of clusters. They used K-means, K-model clustering, and fuzzy C-means to predict thyroid illness. They expanded the number of clusters in the thyroid dataset and discovered that their method is more efficient than others at clustering. The authors (Chen et al., 2017) defined a method for diagnosing thyroid disease during pregnancy. The deep learning algorithm is used in their proposed solution, which achieves 98.22% accuracy. Chaurasia, Pal & Tiwari (2018) analyzed data using a variety of machine learning algorithms. They obtained 97.37 percent accuracy using Naive Bayes.

Aversano et al. (2021) analyzed data using a variety of machine learning algorithms. They compared the output of ten different classifiers in particular. The performance of the other algorithms is encouraging, particularly the Extra-TreeClassifier, which achieves an accuracy of 84%. Additionally, they employed a catboost classifier and obtained a precision of 71%. Kumar (2020) detected thyroid stage with an accuracy of 83.37% using SVM. Other classification algorithms were discovered to be less effective than Multiclass SVM. Additionally, the model correctly distinguishes between the four thyroid states.

The above discussion shows that ANN, CatBoost, XGBoost, Random Forest, LightGBM, Decision Trees and Extra Trees, as well as SVC, KNN and Naive Bayes perform better on many thyroid multiclass datasets. These algorithms outperform all others in terms of accuracy and overall performance.

### Dataset overview

We chose the Sick-euthyroid dataset (Quinlan, 1987) above the other ten due to its size and class. Increased data availability enables us to develop new and more sophisticated insights and applications. The dataset has 3,162 rows and 25 data columns. Consider the other datasets in allbp.data, which include only 2,800 instances and no missing values. Additionally, allhyper.data contains only 2,800 instances and contains no missing values. Each data set and test set contain the same 2,800 instances and 972 cases. However, not just because of the volume of cases, we discover that relatively few researchers have previously worked with this dataset. As a result, we concentrated on this dataset and studied the results in order to aid future researchers in predicting similar types of multiclass thyroid datasets.

The Sick-euthyroid dataset is available in the UCI machine learning repository (Dua & Graff, 2017). The dataset can be found here: https://archive.ics.uci.edu/ml/datasets/thyroid+disease. The dataset is composed of two files: sick-euthyroid.names and sick-euthyroid.data. Sick-euthyroid.names is a file that contains the names of the attributes and any associated data. The other data file contains the database’s metadata, as well as the classes, attribute names, and any other data that may be present. The total number of rows in the dataset is 3,162, with 25 data columns. Table 1 outlines the data set’s general structure.

**Table 1:** Sick-euthyroid dataset structure.

Column	Value	Value	Missing
age	continuous		Y
sex	M	F	Y
on_thyroxine	f	t	N
query_on_thyroxine	f	t	N
on_antithyroid_medication	f	t	N
thyroid_surgery	f	t	N
query_hypothyroid	f	t	N
query_hyperthyroid	f	t	N
pregnant	f	t	N
sick	f	t	N
tumor	f	t	N
lithium	f	t	N
goitre	f	t	N
TSH_measured	n	y	N
TSH	continuous		Y
T3_measured	n	y	N
T3	continuous		Y
TT4_measured	n	y	N
TT4	continuous		Y
T4U_measured	n	y	N
T4U	continuous		Y
FTI_measured	n	y	N
FTI	continuous		Y
TBG_measured	n	y	N
TBG	continuous		Y

In a classification problem for predictive modeling, two class labels can be used. This is called binary classification or two-class classification. Since the sick-euthyroid has only two classes - sick-euthyroid and negative, it indicates that it is a binary classification problem. Furthermore, the distribution of the classes is not equal in this classification predictive modeling problem. There are 2,870 cases in this dataset that fall into the sick-euthyroid category and 292 instances that fall into the negative category; these are the only instances that compose our training dataset. This is an illustration of an imbalanced classification problem. Under-sampling and over-sampling are two methods for creating a balanced dataset from an imbalanced one.

• **Under-sampling:** By lowering the size of the abundant class, under-sampling balances the dataset. When the amount of data is sufficient, this method is utilized. By retaining all samples in the uncommon class and randomly selecting an equal number of samples from the abundant class, a new balanced dataset suitable for further modeling can be retrieved (Liu, Wu & Zhou, 2008).
• **Over-sampling:** When the amount of data is insufficient, oversampling is used. It attempts to balance the dataset by enlarging unusual samples (Zhu, Lin & Liu, 2017). Furthermore, instead of discarding abundant instances, new unique data are drawn by the use of techniques such as recurrence, bootstrapping, or SMOTE (Synthetic Minority Over-Sampling Technique) (Chawla et al., 2002).

Oversampling the minority class is one approach for dealing with imbalanced data in the thyroid dataset. Even if the duplicated instances contribute no additional information to the model, the simplest strategy is to duplicate instances from the minority class. Rather than that, new examples can be synthesized from current ones (Han, Wang & Mao, 2005). This technique is referred to as the Synthetic Minority Oversampling Technique or SMOTE for short, and it is used to augment data for the minority class (Chawla et al., 2002). This research makes use of the SMOTE class implementation provided by the imbalanced-learn Python library. Similar to a scikit-learn data transform object, the SMOTE class must be defined and configured prior to being fitted to a dataset and applied to create a new modified version of the dataset. To begin, we use SMOTE to oversample the minority class, which means we may alter the example to oversample the minority class to contain 10% of the instances of the majority class. We can pass the desired ratios to the SMOTE and RandomUnderSampler classes as parameters. Following that, we may combine these two transformations into a pipeline. The pipeline can then be applied to a dataset, doing each transformation in turn and delivering a final dataset that contains the aggregate of the transformations performed.

### Algorithms overview

Generally, it is accepted that having insufficient training data leads to a poor approximation. For our study problem, we used vanilla neural networks. However, we realized that we do not have sufficient data to learn the mapping function or evaluate models in practice. We require an adequate number of cases to obtain a valid approximation to the unknown underlying mapping function. The data in our dataset is insufficient for deep learning models. That is why, rather than using existing deep learning models, we chose the different tree and statistical models to give an overall comparison of this research work. For this study, several machine learning algorithms are studied and used for the comparison task. The basic working mechanisms of the utilized algorithms are discussed in this section.

#### Decision tree

The decision tree technique is a subset of supervised machine learning that is based on a continuous data splitting mechanism across specified parameters (Rokach, 2016). Internal nodes reflect attributes, branches represent decision laws, and each leaf node in the recursive decision tree algorithm interprets the outcome. The data is categorized using the Top-Down approach, which involves sorting from the root to a leaf/terminal node. After obtaining the training data, it splits the dataset into small subsets using the Gini Index, Information Gain, Entropy, Gain Ratio, and Chi-Square. The Gini Index and Information Gain are measured in the majority of datasets using Eqs. (1) and (2) respectively. The process is repeated for each child tuple until all tuples belong to the same class and no additional attributes are needed. The Gini Index is used to increase precision and diagnostic accuracy.

(1) \begin{eqnarray*}Information~Gain=Entropy(S)[(Weighted~Avg)\ast Entropy(each~feature)].\end{eqnarray*}

(2) \begin{eqnarray*}Gini~Index~=~1~-~\sum _{j=1}^{c} \frac{}{} {\mathbf{P}}_{j}^{2}.\end{eqnarray*}

Here in Eq. (2), P_j_ is proportion of the samples that belongs to class c for a particular node.

This algorithm traverses the potential decision-making space using a top-down greedy search strategy, never retracing and reconsidering prior choices (Jin, De-Lin & Fen-Xiang, 2009). The metric used in this algorithm to evaluate the best attribute at each point of the expanding tree is precisely information gain. To begin, a predictive model must be trained using available data and then validated for accuracy and reliability using test data collected specifically for the purpose of predicting proven test outcomes (Song & Ying, 2015). To evaluate the optimal attribute for breaking records using Attribute Selection Measures (ASM), we may employ various methods, including the Gini index or information gain(Tangirala, 2020). This attribute then subdivides the dataset into smaller subsets for each child until there are no more attributes.

**Figure 1:**
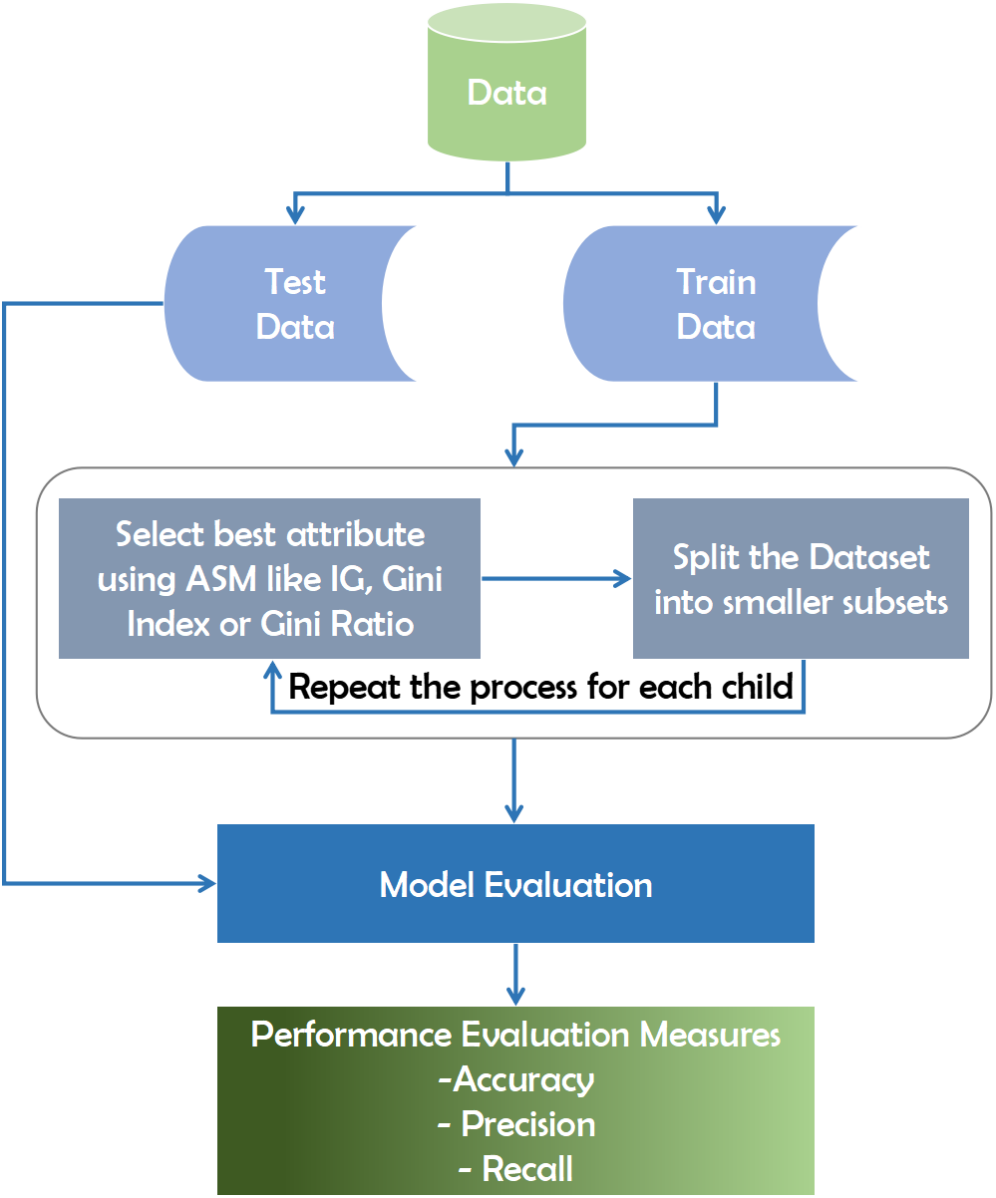
Working mechanism of decision tree technique.

As shown in the diagram, the model is initially created using training data. The model’s accuracy is determined, and it is optimized by estimating the known outcome using the data. Finally, the model can be used to make predictions about future outcomes. Predictive ratings would be calculated after the computer program has constructed the forest. The predictive score is calculated as a percentage of the objective predictor in the eligible model’s terminal node (or leaf). We can use various strategies such as the Gini index or knowledge gain to determine the best attribute to break the records using Attribute Selection Measures (ASM). This attribute then subdivides the dataset into smaller subsets for each child until no more attributes remain. Figure 1 depicts the working mechanism of the decision tree.

#### Random forest

A random forest is composed of multiple independent decision trees that function in combination (Liaw et al., 2002). Each tree produces *ad hoc* data samples. Based on the votes, it determines the best prediction score. Additionally, it identifies significant elements within a dataset and provides a simple indicator of the feature’s significance. Feature selection is often used in classification studies to reconstruct the data and improve accuracy. The feature selection technique is applied in a number of ways, including filtering (Filter) and encapsulation(Wrapper). The filtering method’s feature selection function was independent of the classification algorithm and was based on a feature of the data set. The utility of a function contributes to its accuracy (Lebedev et al., 2014). A random forest can yield more reliable ensemble forecasts than a decision tree, which is the primary distinction between the two. The F value of a test statistic for a single function is calculated by the feature selection procedure, which follows the Equation (3).

(3) \begin{eqnarray*}norm~f{i}_{i}= \frac{f{i}_{i}}{{{\Sigma }_{i}}_{j\in all~features}fi} .\end{eqnarray*}

Here in Eq. (3), normfi_i_ is the normalized importance of feature i, fi_i_ represents the importance of feature i.

Following that, the total number of trees is divided by the value assigned to each node’s feature’s importance utilizing Eq. (4).

(4) \begin{eqnarray*}RFf{i}_{i}= \frac{{\Sigma }_{j\in alltrees}normf{i}_{ij}}{T} \end{eqnarray*}

Here in Equation (4), RFfi_i_ the importance of feature i calculated from all trees in the Random Forest model, normfi_ij_ represents the normalized feature importance for i in tree j, and T is the total number of trees.

**Figure 2:**
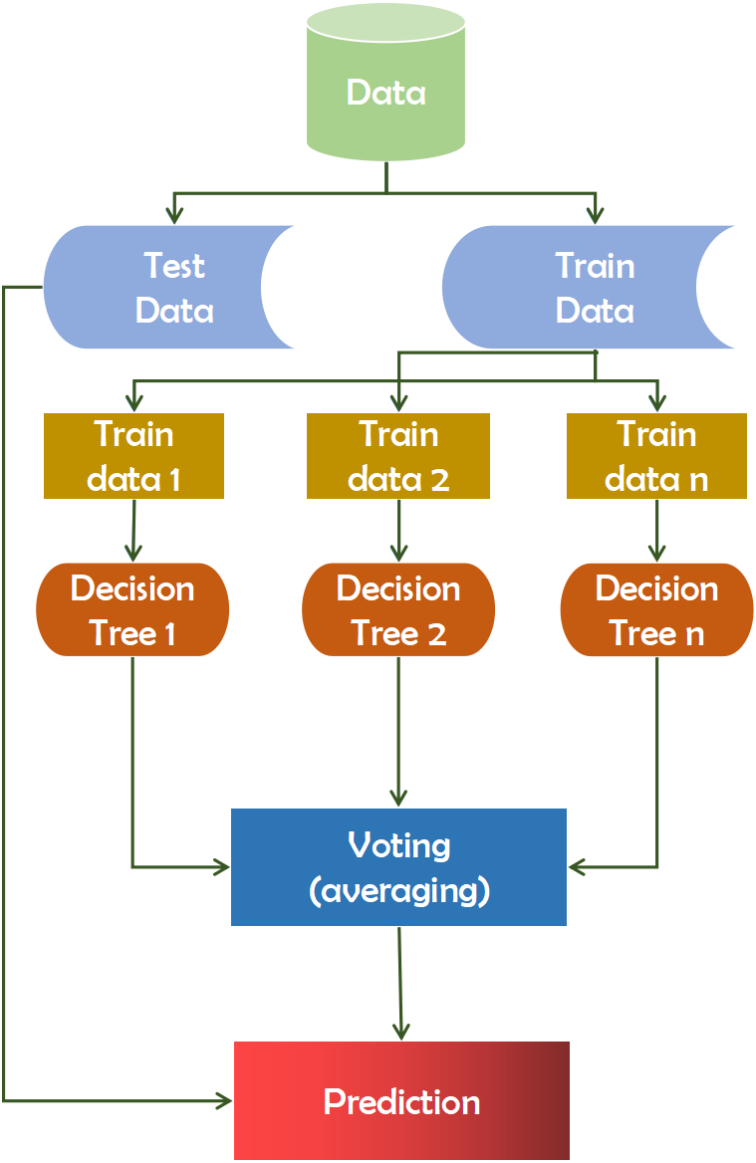
Working mechanism of random forest technique.

This technique extracted data points from the training set and constructed trees for each data point. Each decision tree generates a prediction result during the training phase. When a new data point is acquired, the Random Forest classifier predicts the ultimate decision based on the majority of products (Ali et al., 2012). Recent computational biology work has witnessed a growth in the usage of Random Forest, owing to its inherent benefits in dealing with tiny sample numbers, a high-dimensional feature space, and complex data structures (Qi, 2012). Using the random forest has a good chance of improving accuracy and assisting significantly in diagnostic cases. Figure 2 depicts the working mechanism of the random forest technique.

#### LightGBM

LightGBM is a gradient boosting technology built on top of a tree-based learning algorithm. It performs at a rate six times faster than the XGBoost algorithm (Wang, Zhang & Zhao, 2017). The algorithm is called “light” due to its computational power and ability to produce faster results. Unlike other tree-based algorithms, the LightGBM tree grows vertically, or leaf by leaf (Ke et al., 2017). Due to the dispersion of high-dimensional data, it is necessary to create a virtually lossless path in order to reduce the number of features. As a result, the training phase can be completed more quickly while keeping the same level of accuracy. As an outcome, diagnostic accuracy would ultimately improve. The XGBoost algorithm, which is based on the GBDT technique and the preceding definition, incorporates the regular function to prevent the model from becoming too well-fit. The decision tree model splits each node at the most informative feature (with the largest information gain). For GBDT, the information gain is usually measured by the variance after splitting, which is defined as below (Ke et al., 2017). Equation (5) is widely utilized in machine learning at present.

(5) \begin{eqnarray*}{{\vee }_{j\mid }}_{\mathcal{O}}(d)=( \frac{1}{{n}_{o}} \frac{({\Sigma }_{{x}_{i}\in \mathcal{O}:{x}_{ij\leq d}}{g}_{i})^{2}}{{n}_{{l{|}\mathcal{O}}^{j}}(d)} + \frac{({\Sigma }_{{x}_{i}\in \mathcal{O}:{x}_{ij\succ d}}{g}_{i})^{2}}{{n}_{r{|}\mathcal{O}}^{j}(d)} ).\end{eqnarray*}

In Eq. (5), $\mathcal{O}$ represents the training on a fixed node of the decision tree. ${{&or; }_{j&mid; }}_{\mathcal{O}}(d)$ is the variance gain of splitting feature *j* at point *d* for this node (Ke et al., 2017). For feature *j*, the decision tree algorithm selects ${d}_{j}^{\ast }=~argma{x}_{d}{V}_{j}(d)$ and calculates the largest gain ${V}_{j}({d}_{j}^{\ast })$. Then, the data are split according feature *j*^∗^ at point ${d}_{j}^{\ast }$ into the left and right child nodes (Ke et al., 2017).

**Figure 3:**
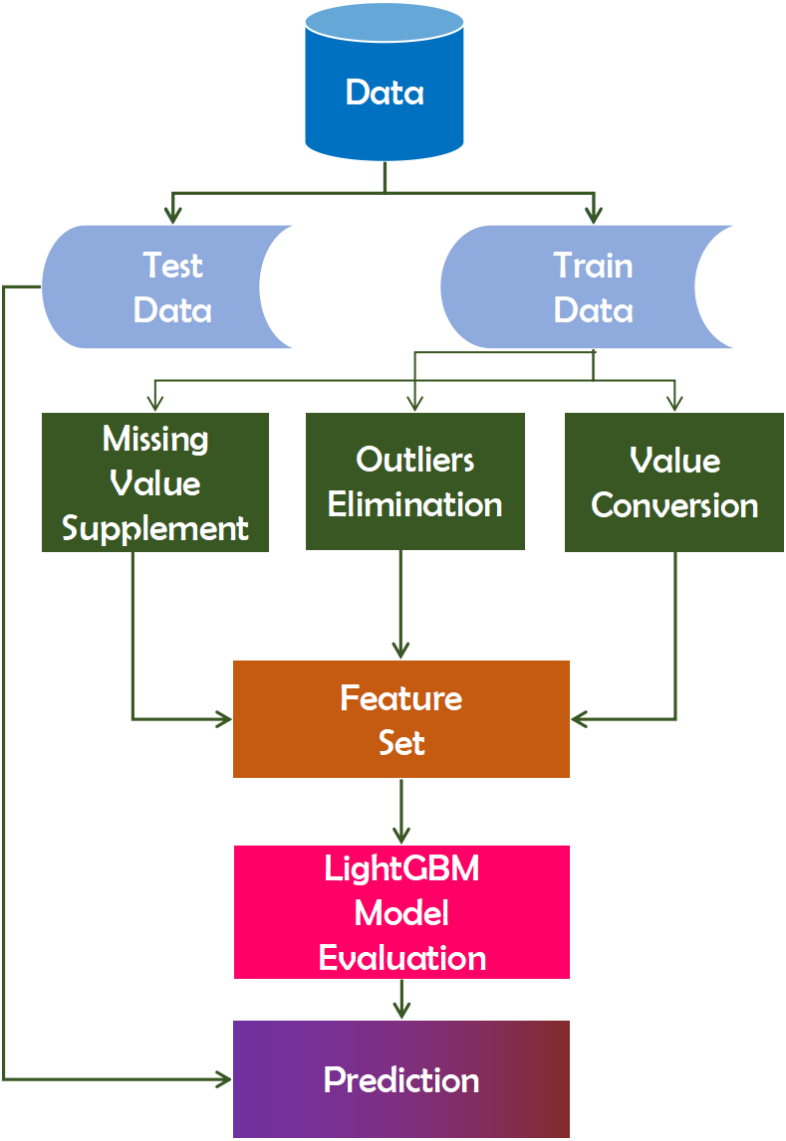
Working mechanism of LightGBM technique.

This Eq. (5) can be utilized to define the algorithm’s regular term. This is a more precise definition that the algorithm inventor arrived at after conducting numerous experiments, and researchers can use it directly (Ma et al., 2018). This algorithm runs on the dataset, followed by pre-processing the training data for pre-training. The pre-processed data is then classified according to the importance of the features. This analysis will aid in our comprehension of the data (Ju et al., 2019). Figure 3 depicts the working mechanism of the LightGBM technique.

#### XGBoost

XGBoost is an approach that utilizes ensembles. Ensemble learning is a method for combining the predictive abilities of numerous learners in a systematic manner. As a result, a consolidated model is built from the output of multiple models. The ensemble’s models, which are occasionally referred to as fundamental learners, may originate from the same or different learning algorithms (Li, Fu & Li, 2020). If a dataset has n instances, which corresponds to n rows, we use i to represent each instance. The XGboost algorithm, based on the GBDT technique and the preceding definition, introduces the regular function Ω(*f*) to control the model’s over-fitting.

(6) \begin{eqnarray*}\min \nolimits \{ obj\} ~=\min \nolimits \{ L({F}_{m}(X),Y)+\Omega (f)+C\} .\end{eqnarray*}

Here, *L*(*F*_*m*_(*X*), *Y*) represents the Loss function. The difference between the final predicted variables after *m* times iteration and the actual ones, Ω(*f*) indicates the regularization function in the final prediction model after all iterations.

Equation (7) is a more precise form that the algorithm designer derived after conducting numerous trials, and researchers can immediately apply it Ma et al. (2018).

(7) \begin{eqnarray*}\Omega ({f}_{t})=\gamma T+ \frac{1}{2} \lambda \sum _{j=1}^{T}{\omega }_{j}^{2}.\end{eqnarray*}

In Eq. (7) the regular item function is a function of leaf number *T*, and leaf node output result *ω*_*j*_.Ω(*f*_*t*_) represents the the function of regularization in the *t* wheel iteration.

**Figure 4:**
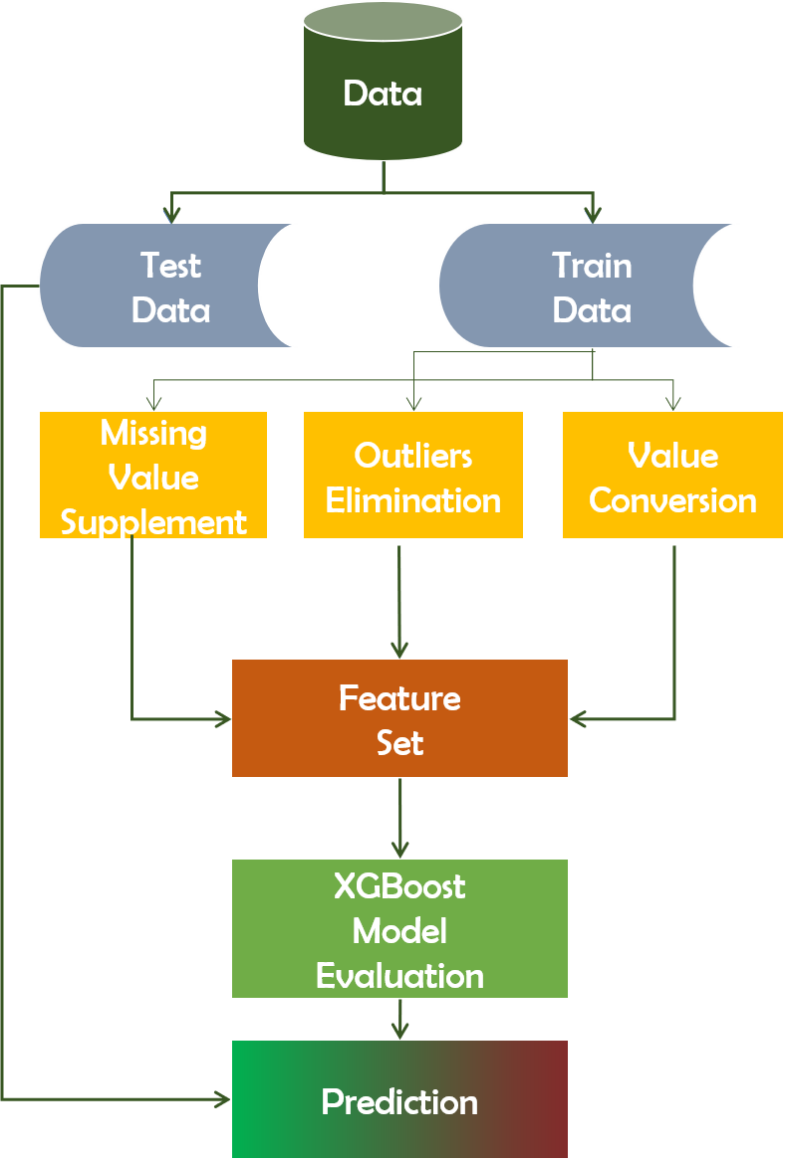
Working mechanism of XGBoost technique.

It automatically handles missing data values and tracks structured databases when dealing with classification and regression predictive modeling issues. XGBoost predicts residuals or errors of previous models (Chen et al., 2015), combined in order to generate the last forecast. It also improves efficiency by reducing the over-fitting of data sets. Figure 4 depicts the working mechanism of the XGBoost technique.

#### GaussianNB

The technique of Gaussian Naive Bayes (GaussianNB) is predicated on the susceptibility to predictor independence (Rish et al., 2001). A classifier of GaussianNB, in general, means that a function is not linked to any other variable in a class. It is easy to make for large data sets and particularly useful. As each feature is independent, the inclusion of one feature does not affect the appearance of other features in the GaussianNB algorithm. The algorithm is based on the Bayes theorem, and the probability can be calculated utilizing Equation (8).

(8) \begin{eqnarray*}P(A{|}B)= \frac{P(B{|}A)P(A)}{P(B)} \end{eqnarray*}

**Figure 5:**
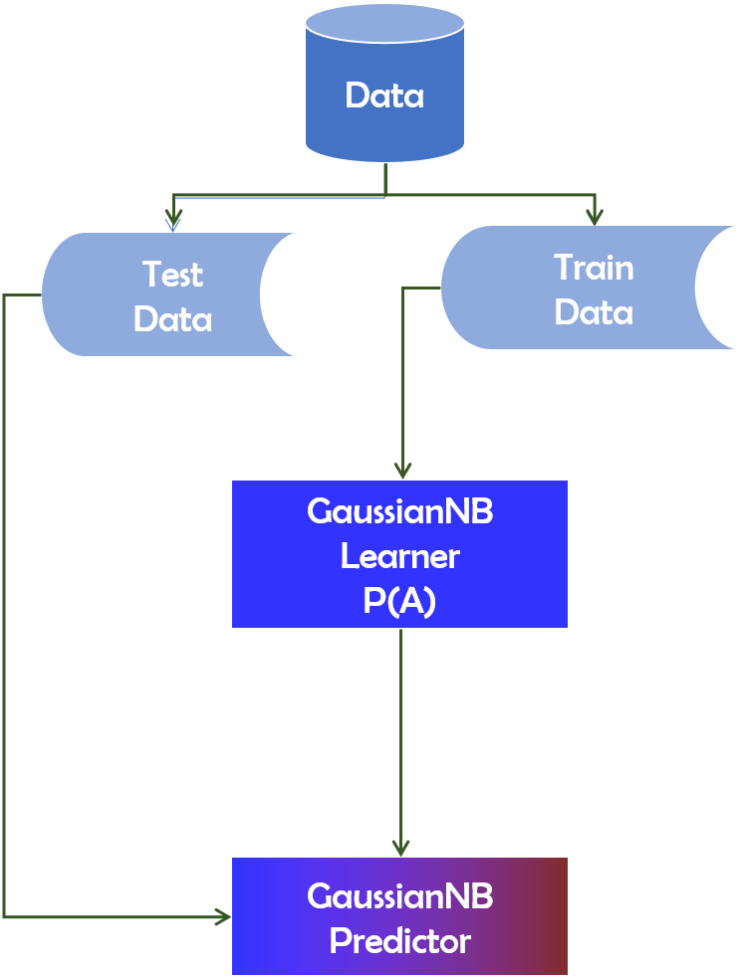
GaussianNB technique’s operational mechanism.

The probability *P*(*A*|*B*) that we are interested in computing is referred to as the posterior probability. *P*(*A*) is the probability of occurrence of event *A*. Similarly, *P*(*B*) is the probability of occurrence of event *B*.*P*(*B*|*A*) denotes the conditional probability of event B occurring if A occurs. Figure 5 depicts the working mechanism of the GaussianNB technique.

#### KNN

**Figure 6:**
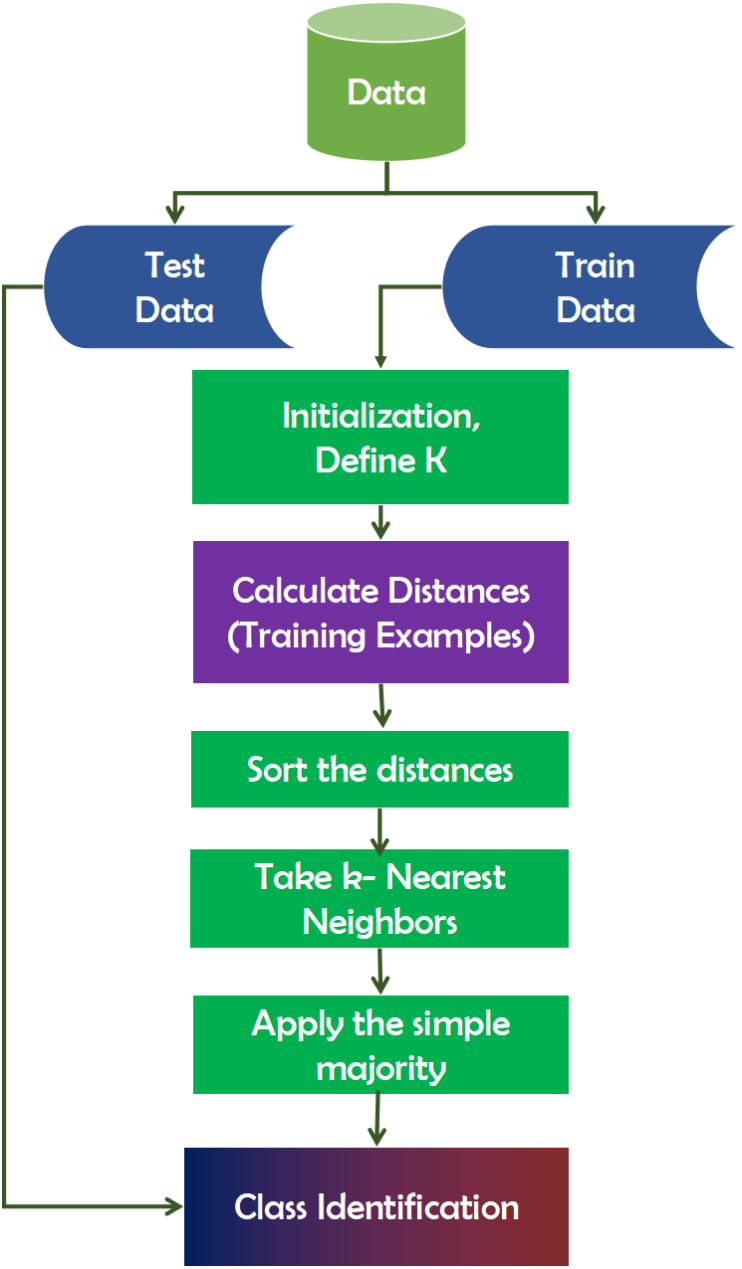
Working mechanism of KNN technique.

K-Nearest Neighbors (KNN) is a technique for inducing laziness in learning (Gou et al., 2012). That is to say, it makes no assumptions about the distribution of the data on which it is based. Frequently, the model is evaluated using the dataset. It is beneficial when working with real-world datasets. Additionally, there are no training data points needed for model generation. All training data is incorporated into the testing process. While this expedites planning, it delays testing and necessitates a great deal of time and memory. When building the model, the number of neighbors (K) must be specified in KNN. In this case, K acts as a controlling variable for the prediction model. When the number of classes is even, K is usually an odd number. It does not immediately learn from the training set; rather, it maintains the dataset and uses it for classification. Figure 6 depicts the working mechanism of the KNN technique.

#### ANN

Artificial Neural Networks (ANN) is designed to replicate human studies by modeling human brain structures (Dongare et al., 2012). There are input and output layers in neural networks, as well as a hidden layer that contains components that convert the input to a format that the output layer can use. Three interconnected layers comprise ANNs. It is also known as a Feed-Forward Neural Network (Benardos & Vosniakos, 2007).

**Figure 7:**
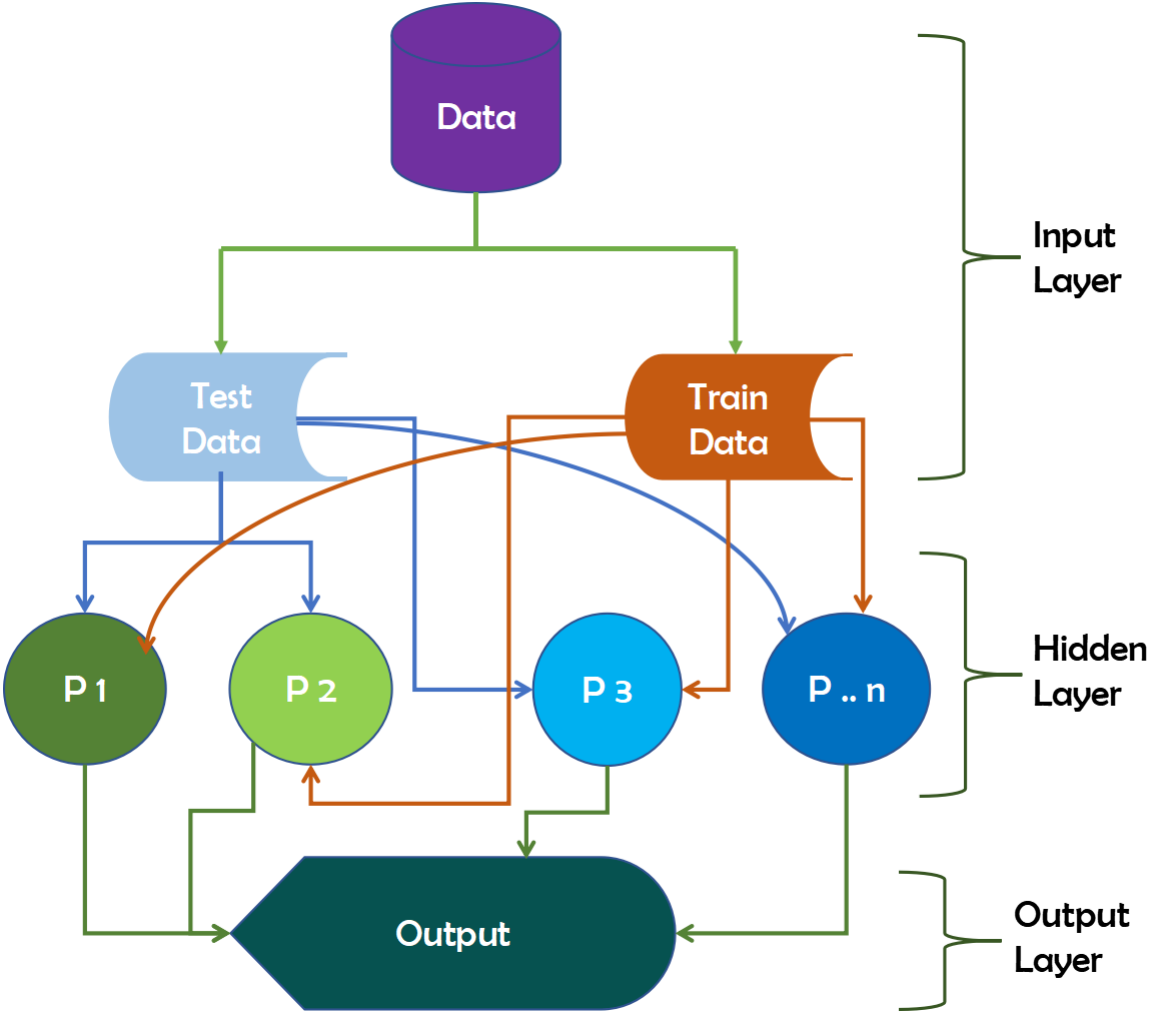
Working mechanism of ANN technique.

The input neurons receive the data that we feed the ANN in the input layer. These neurons transmit data to the hidden layer, which performs the magic, and then transfer output neurons to the output layer, which stores the network’s final calculations for future use. The data will produce outputs with insufficient data after ANN training. It can also learn on its own and provide results. Figure 7 depicts the working mechanism of the ANN technique.

#### SVC

**Figure 8:**
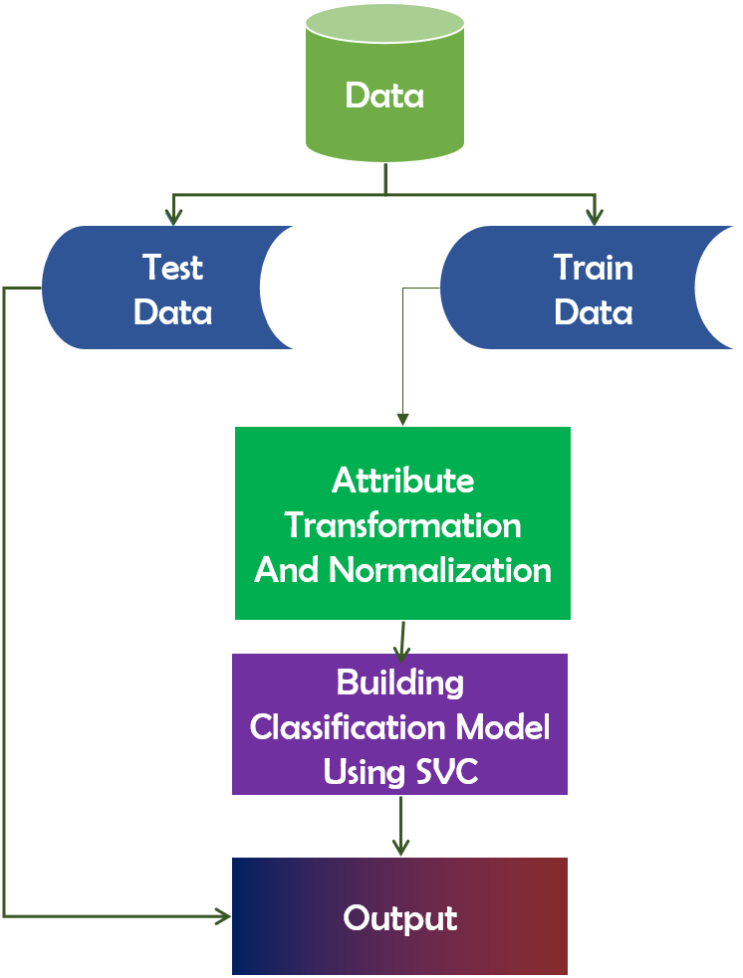
Working mechanism of support vector classifier.

In the features that categorize data points, the Support Vector Classifier (SVC) algorithm functions as a hyperplane (Rueping, 2010). Maximizing the data point distance margin reinforces the need for future data point organization. The distinction between SVC and SVM is that LinearSVC is defined in terms of liblinear, whereas SVC is defined in terms of libsvm. That is why LinearSVC offers greater flexibility in terms of penalty and loss function selection. Additionally, it scales better to large sample sizes. It is a technique for determining the fittest separation hyperplane. The hyperplane has the greatest margin gap between the two groups’ nearest points. This approach ensures that the larger the margin, the lower the classifier’s generalization error. Figure 8 depicts the framework of SVC.

#### CatBoost

**Figure 9:**
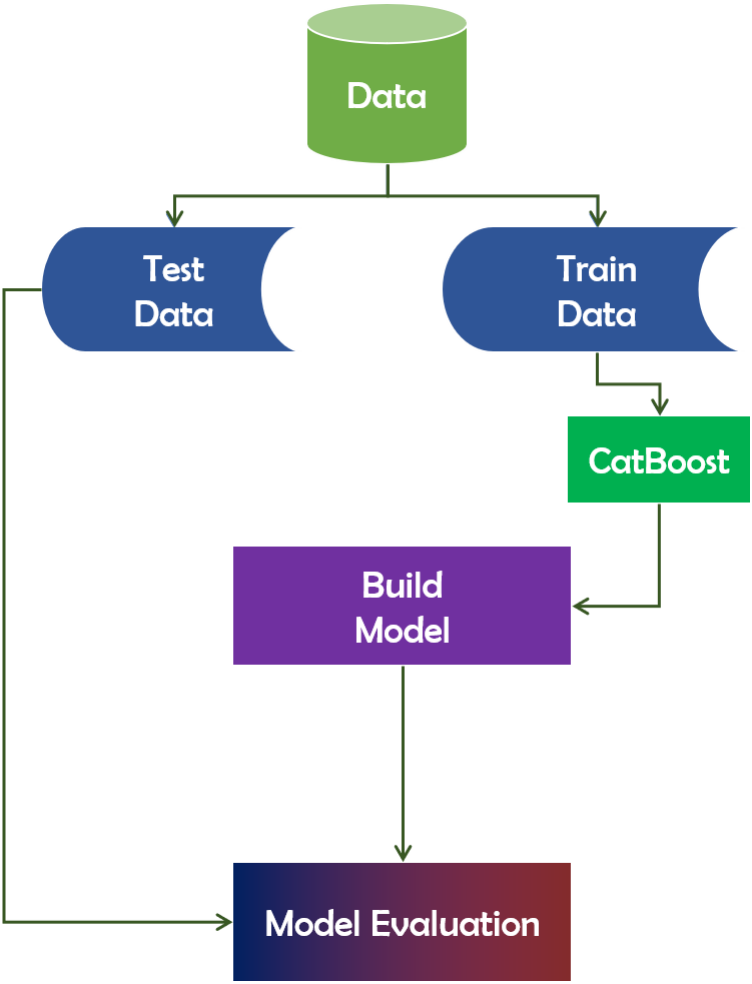
CatBoost algorithm’s working mechanism.

The name “CatBoost” is derived from the phrases “Category” and “Boosting”. CatBoost supports numerical, categorical, and text features but excels at categorical data handling (Dorogush, Ershov & Gulin, 2018). It is a publicly available machine learning algorithm. It is based on the gradient boosting library. Additionally, it is capable of producing outstanding outcomes with a minimal amount of data, as opposed to deep learning models, which require a vast amount of data to train from. In terms of performance, CatBoost vastly beats all other machine learning algorithms. Additionally, CatBoost can be used to convert categories to numbers without conducting any explicit pre-processing. Figure 9 depicts the working mechanism of the CatBoost technique.

#### Extra-Trees

**Figure 10:**
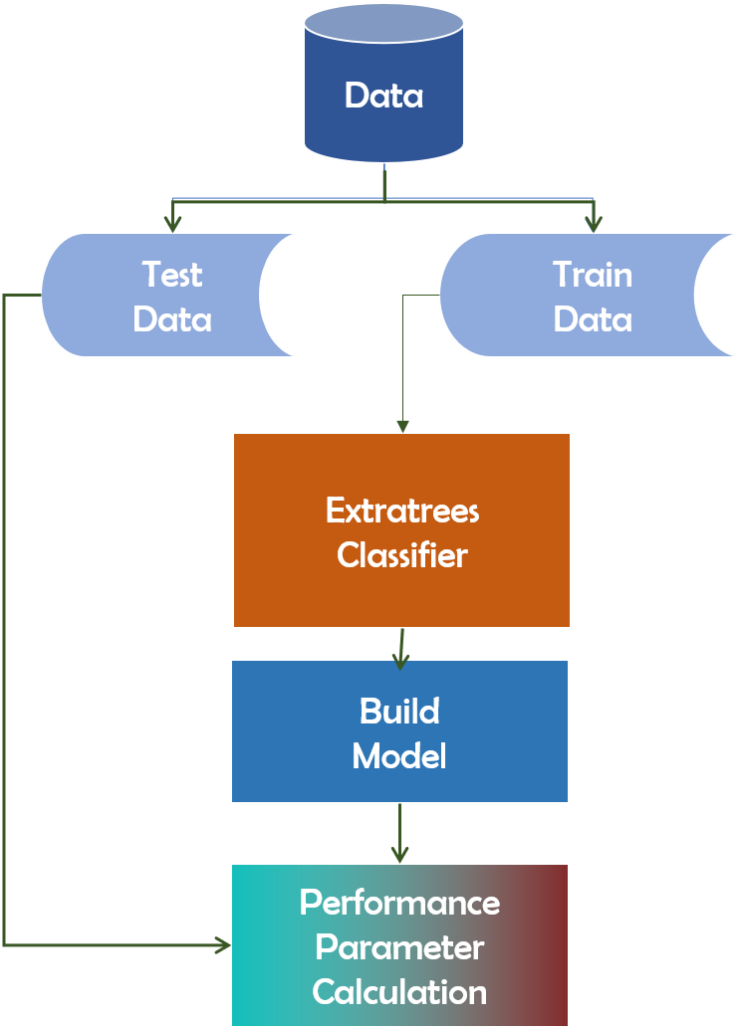
Extra-Trees algorithm’s working mechanism.

The Extra-Trees classifier is a decision tree-based ensemble learning approach. Extra-Trees classifier randomly selects specific decisions and subsets of data in order to avoid overlearning and overfitting. The Extra-Trees classifier is a decision tree-based ensemble learning approach (Bhati & Rai, 2020). Extra-Trees classifier randomly selects specific decisions and subsets of data in order to avoid overlearning and overfitting. It creates several trees and separates nodes based on random feature subsets. Randomness is introduced into Extra-Trees not by data bootstrapping but through random splits of all observations. Figure 10 depicts the framework of the Extra-Trees classifier.

## Methodology

Data gathering is the initial stage of this research. Choosing which data gathering strategy to utilize depends on our overall study aims, objectives, practicality, and resource restrictions. The gathered data is next evaluated to prepare it for the model selection procedure. Data preprocessing is a data mining approach to transform the raw data acquired from varied sources into cleaner information that’s more suited for work. Raw data might contain missing or inconsistent values and provide a lot of duplicate information. In this data processing stage, we may verify whether there is any data missing or contains erroneous data and outliers that we can identify in the data set or check the absence of data limitations. The absence of data frequently leads to inconsistent data that causes variations that we have to deal with before analysis. Then the data is separated into train and test datasets.

**Figure 11:**
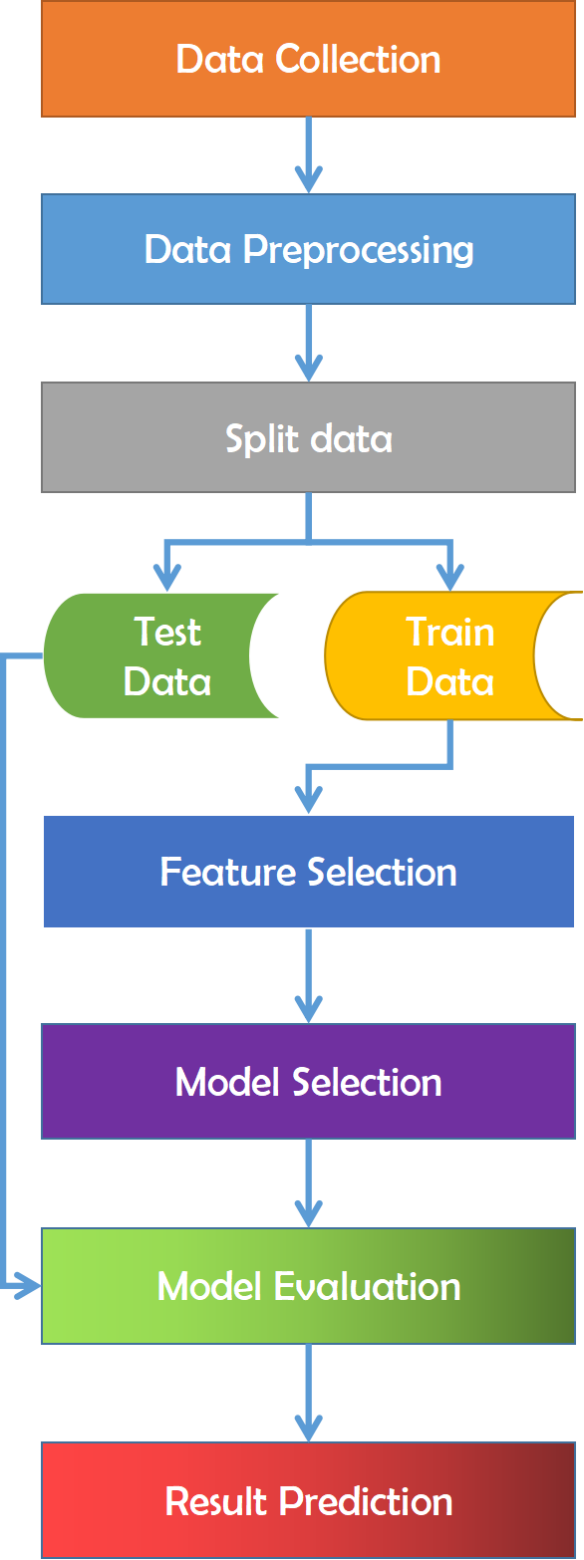
Overview of the employed methodology for this study.

The train-test split is a strategy for analyzing the performance of a machine learning system. It may be used for classification or regression issues and can be utilized for any supervised learning strategy. The process includes taking a dataset and separating it into two subgroups. The first subset is used to fit the model and is referred to as the training dataset. The second subset is not used to train the model; instead, the input element of the dataset is presented to the model, then predictions are generated and compared to the predicted values. This second dataset is referred to as the test dataset. The purpose is to assess the machine learning model’s performance on new data: data not used to train the model. After that, a feature selection procedure is carried out. Feature selection is the process of limiting the amount of input variables while creating a predictive model. It is desired to limit the number of input variables to both lower the computing cost of modeling and, in some situations, increase the model’s performance. It assesses the relationship between each input variable and the target variable using statistics and selects those input variables that have the most essential association with the target variable. It is necessary for classification that the dataset comprises only numerical characteristics; consequently, we must encode the category values into numerical values. Finally, the processed data is utilized to implement all of the machine learning algorithms Fig. 11 refers to the methodology that has been applied for this work.

### Data collection

The Sick-euthyroid dataset used in this work was available from the University of California, Irvine’s machine learning repository (Dua & Graff, 2017). The dataset consists of 3,162 rows and 25 columns of data. This dataset is sufficiently rich to allow us to interact with it and see some frequent behaviors. This dataset contains 25 characteristics, with 2,870 samples classified as negative and 292 as sick-euthyroid. The section Dataset Overview discusses this dataset in further detail.

### Data preprocessing

We observed the set of potential values for each characteristic at the start of data preprocessing. As we saw that the ‘TBG’ column includes 91.78% missing values, we removed this column. For the rest of the missing values in different columns, fillna method (Pandas development team, 2021) is used to substitute the missing values with the mean and median.Then we transformed the object to an int by substituting the following values: “t”:1, “f”:0, “y”:1, “n”:0, “sick-euthyroid”:1, “negative”:0, “F”:1, “M”:0.

**Figure 12:**
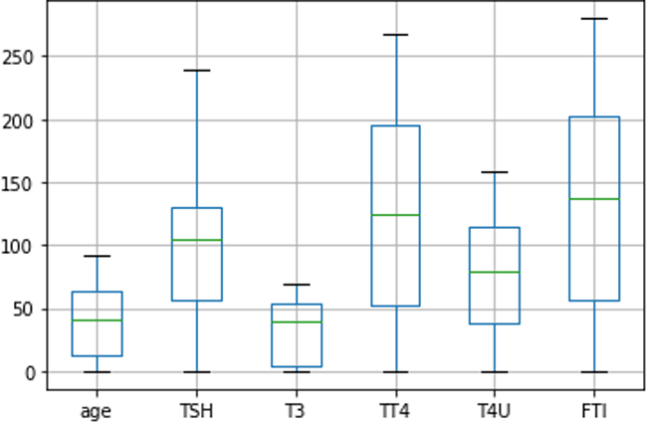
Distribution of age, TSH, T3, TT4, T4U, FTI data using boxplot diagram.

Among the processed dataset, 80%, 2530 rows are used to train the models, and 20%, 632 rows are used to evaluate the model’s performance. Then, we utilized a boxplot Fig. 12 to illustrate the form of the distribution, its central value, and the column ‘age’, ‘TSH’, ‘T3’, ‘TT4’, ‘T4U’, ‘FTI’ variability.

### Feature selection

Feature selection techniques assign a rating to input features based on their predictive ability for a target variable. Based on the Fig. 2 feature selection score, necessary features are selected. Scores for feature selection are critical for optimizing the response of a predictive model. It prioritizes the attributes that contribute to the predicted variable’s accuracy and eliminates those contributing to the model’s inaccuracy.

**Figure 13:**
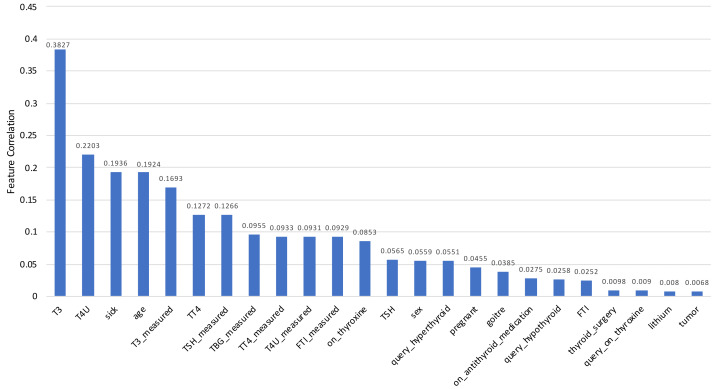
Correlation scores for every feature of the Sick-euthyroid dataset is plotted in a bar chart. Each column represents the score for for relevant feature. The scores are sorted in descending order.

To measure this feature selection score, scikit-learn (Scikit-learn developers, 2021) implementation of principal component analysis (PCA) is used in this study. The model includes a feature importance property which is used to retrieve the relative importance scores for each input function after it has been fitted into the model. Feature importance scores for each feature of the dataset are placed in Table 2, and the visual representation is depicted in Fig. 13 for a better understanding. Feature importance scores are laid out in descending order, providing the most important feature in the first place and the least important feature in the last place.

**Table 2:** Selecting features based on correlation.

Feature name	Correlation score
T3	0.3827
T4U	0.2203
sick	0.1936
age	0.1924
T3_measured	0.1693
TT4	0.1272
TSH_measured	0.1266
TBG_measured	0.0955
TT4_measured	0.0933
T4U_measured	0.0931
FTI_measured	0.0929
on_thyroxine	0.0853
TSH	0.0565
sex	0.0559
query_hyperthyroid	0.0551
pregnant	0.0455
goitre	0.0385
on_antithyroid_medication	0.0275
query_hypothyroid	0.0258
FTI	0.0252
thyroid_surgery	0.0098
query_on_thyroxine	0.0090
lithium	0.0080
tumor	0.0068

The Feature Selection Score is crucial in the situation of multicollinearity. It occurs when one predictor variable in a multiple regression model can be accurately predicted linearly from the others, resulting in skewed or misleading results. That is the primary reason for removing those features prior to training the model.

### Performance metrics

Metrics are used to monitor and quantify a model’s performance. Various performance indicators are used to compare various Machine Learning Algorithms. For the purposes of our research, we will concentrate on those that are utilized to solve classification difficulties. We can utilize performance measures for categorization such as Accuracy, Precision, Recall, and F1-score.

**Accuracy:** In classification problems, accuracy refers to the number of correct predictions made by the model over all possible predictions. It is calculated by multiplying the number of correct predictions by the total number of predictions multiplied by 100.

(9) \begin{eqnarray*}Accuracy~=~ \frac{{T}_{P}+{T}_{N}}{{T}_{P}+{F}_{P}+{F}_{N}+{T}_{N}} .\end{eqnarray*}

Here, *T*_*P*_ is True Positive, where a person is actually having euthyroid sick syndrome(1), and the model classifying his case as sick-euthyroid(1) comes under True Positive. *T*_*N*_ is True Negative, where a person not having euthyroid sick syndrome(0) and the model classifying his case as Negative(0) comes under True Negatives. *F*_*P*_ is False Positive, where a person NOT having Euthyroid sick syndrome(0) and the model classifying his case as sick-euthyroid(1) comes under False Positives. *F*_*N*_ is False Negative, where a person having Euthyroid sick syndrome(1) and the model classifying his case as Negative(0) comes under False Negatives.

**Precision:** Precision is the ratio of true positives and total positives predicted.

(10) \begin{eqnarray*}Precision~=~ \frac{{T}_{P}}{{T}_{P}+{F}_{P}} .\end{eqnarray*}

Here, precision is a measure that tells us what proportion of patients that diagnosed as having euthyroid sick syndrome, actually had the euthyroid sick syndrome. The predicted positives (People predicted as sick-euthyroid are *T*_*P*_ and *F*_*P*_) and the people actually having a euthyroid sick syndrome are *T*_*P*_.

**Recall:** A recall is the ratio of true positives to all positives in the ground truth.

(11) \begin{eqnarray*}Recall~=~ \frac{{T}_{P}}{{T}_{P}+{F}_{n}} .\end{eqnarray*}

Here, recall is a metric that indicates the proportion of individuals who truly had euthyroid sick syndrome who were diagnosed as sick-euthyroid by the algorithm. The actual positives (those with the euthyroid sick syndrome are TP and FN), as well as the patients diagnosed with euthyroid sick syndrome by the model, are TP. FN is included since the Person did indeed have a euthyroid sick syndrome, despite the model’s prediction.

**F1-score:** Precision and recall are combined in the F1-score metric. Indeed, the F1 score is the mean of the two harmonics. A high F1-score indicates both great precision and recall. It has an excellent mix of precision and recall and performs well on issues involving imbalanced classification.

(12) \begin{eqnarray*}{F}_{1}~=~ \frac{2}{ \frac{1}{Precision} + \frac{1}{Recall} } .\end{eqnarray*}

### Machine learning models implementation

To begin, the processed Sick-euthyroid dataset is loaded into the program. Following that, all modules, functions, and objects are configured. Pandas module is utilized to load the data directly from the Machine Learning Repository at the University of California, Irvine (Dua & Graff, 2017). Then different exploratory data analysis techniques are implemented to understand the shape of the data. For example, the total number of rows, columns, classes and distribution of data. Then all the elements are combined into a single document, generated data models, and calculated their accuracy using previously unseen data. Classification predictive modeling is the process of predicting a class label for a given observation. Unbalanced classification problems have a skewed or biased distribution of examples across recognized classes.

Classifications that are not balanced (Zou et al., 2016) provide an issue for predictive modeling because the majority of machine learning methods for classification were created with the assumption of an equal number of samples for each class. As a result, models with low predictive performance emerge, especially for the minority group. This is problematic as, in most circumstances, the minority class is more significant than the majority class, making the problem more susceptible to minority class classification errors than to majority class classification errors. Oversampling the minority class is one approach for reconciling unequal datasets. This technique is called the Synthetic Minority Oversampling Technique, or SMOTE, and it is utilized to enhance minority data.

The dataset is trained first and then tested using multiple models on the entire train and test datasets. The model is then encapsulated in a SelectFromModel instance based on the training dataset’s function importances. This is used to pick elements from the training dataset, train a model with the subset of selected features, and then evaluate the model on the test set using the same feature selection scheme. Utilizing these processes, different accuracy values and learning curves for testing prediction accuracy are obtained for different models. This research’s data and code are available here: https://github.com/saimasharleen/thyroid-dataset.

## Experimental Results and Discussions

At first, we partitioned the datasets into train and test subsets. This technique was used to evaluate the performance of machine learning models. By doing this, our categorization issue was resolved. As we utilized the training dataset to train the 10 machine learning models that we had chosen for our research, the test dataset was used to evaluate the performance of the models.We used one neural network model (ANN), six tree-based models (CatBoost, XGBoost, Random Forest, LightGBM, Decision Tree, and Extra-Trees), and three statistical models (SVC, KNN, and GaussianNB) in this study. Experimental results are scrutinized utilizing accuracy, precision, recall, F1-score, and learning curves. These evaluation matrices are compared for ten classification algorithms. The experimental results are summarized in Table 3. Data in this table are ranked based on the F1-score of the models.

**Table 3:** Accuracy, precision, recall and F1-scores for different classification methods employed in this study. The scores are sorted in descending order based on the F1-score value and ranked them in ascending order.

Method name	Accuracy	Precision	Recall	F1-score	Rank (F1-score)
ANN	0.9587	0.957	0.959	0.957	1
CatBoost	0.9538	0.9550	0.9538	0.9538	2
XGBoost	0.9533	0.9539	0.9533	0.9532	3
Random forest	0.9479	0.9498	0.9479	0.9479	4
LightGBM	0.9456	0.9475	0.9456	0.9455	5
Decision tree	0.9432	0.9446	0.9432	0.9431	6
Extra-trees	0.9107	0.9184	0.9107	0.9102	7
SVC	0.8959	0.8964	0.8959	0.8958	8
KNN	0.8621	0.8710	0.8621	0.8613	9
GaussianNB	0.6509	0.7646	0.6509	0.6089	10

Among 3,162 samples, 292 samples were classified as Sick-euthyroid, while 2,870 samples were classified as negative. Here, we found that most of the data belong to one class. Hence, accuracy score did not provide an appropriate idea for the prediction result. Therefore, we considered both accuracy and precision for the evaluation measure, and we also considered the F1-score to give us a single harmonic mean score that accounted for both precision and recall. We used a variety of machine learning models in our experiment, including neural network models, tree-based models, and statistical models. With the F1 score of 95.7%, the ANN classifier has the highest score of all the classification algorithms we evaluated. The CatBoost classifier algorithm achieved the F1 score of 95.38%, which is very similar to the XGBoost algorithm (95.32%). The Random Forest, LightGBM, Decision Tree, and Extra-Trees are also performed well with the F1-score of greater than 90%. Other algorithms scored less than 90%. The F1 scores for SVC, KNN, and GaussianNB are 89.58%, 86.13%, and 60.89%, respectively.

**Figure 14:**
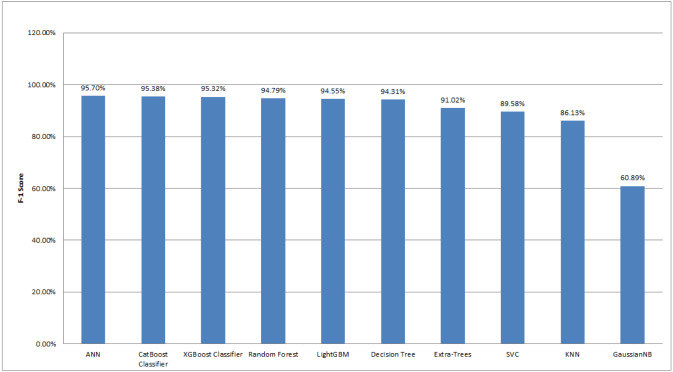
Comparison of F1-scores for different machine learning algorithms employed in this study. The graph is plotted in descending order of the F1-score.

Figure 14 depicts the comparison of the F1 score for different machine learning techniques implemented in this work. After comparing the F1 scores of various algorithms, it is evident that neural networking classifier models performed the best in these types of unbalanced datasets. The primary reason the algorithm performed effectively in our unbalanced dataset because of the boundary between two classes-negative and sick-euthyroid. The decision boundary assisted in determining whether a given data point belonged to a negative or sick-euthyroid class. The activation function was another reason ANN performed well on this dataset. Activation functions endowed the network with nonlinear features. This enabled the network to understand intricate correlations between input and output, as well as if any unbalanced datasets lack an active function.

Now, if we analyze another type of machine learning model, the tree-based model, we found that it outperformed statistical models. We used a variety of tree-based algorithms in our experiment, including CatBoost, XGBoost, Random Forest, LightGBM, Decision Tree, and Extra-Trees. All of these algorithms have F1 scores of more than 90%. The primary reason these algorithms outperformed others in this experiment was that tree-based machine learning algorithms did not require pre-processing features such as standardization or normalization. In contrast, other algorithms, particularly statistical algorithms, required feature scaling to avoid features with an extensive range dominating features with a small range. The second reason was that machine learning models based on trees required bagging and boosting. Among tree-based models, the CatBoost classifier performed the best in this experiment (F1-score: 95.38%). It utilized boosting to increase the model’s accuracy. Unlike bagging, boosting generated models one by one. To begin, the first model was trained on the training data. The second model then learned from the same training dataset and from the previous model’s errors. The third model, like the previous two, learned from the same training dataset and from the prior model’s errors. This process was repeated until a sufficient number of models were constructed. Boosting increased the accuracy since each base model learned from the mistakes of the prior model. XGBoost (F1-score: 95.32%), a classifier based on Extreme Gradient Boosting, was another classifier that was more similar to the CatBoost Classifier. XGBoost performed admirably when overfitting occured, as in our case, where the train and test data were not balanced.

Following that, we observed that Random Forest (F1-score: 94.79%), LightGBM (F1-score: 94.55%), and Decision Tree (F1-score: 94.31%) did better in this experiment. In this experiment, the random forest outperformed the decision tree. Predictions made with ensemble approaches such as Random Forest were typically more accurate than those made with a single model, such as a Decision Tree. Moreover, LightGBM outperformed decision trees because it generated significantly more complicated trees *via* a leaf-wise split technique rather than a level-wise split strategy, which was critical for getting a higher F1-score. Additionally, we specified the max_depth parameter to avoid overfitting issues. Lastly, we used Extra-Trees. While Random Forest and Extra-Trees were remarkably similar ensemble algorithms, Extra-Trees has a little lower F1-score than Random Forest. The distinction was that the random forest algorithm used subsamples of the input data with replacement, but the extra trees algorithm used the entire original sample.

Finally, we examined statistical models such as SVC, KNN, and GaussianNB. SVC handled outliers better than KNN, which was the primary reason it surpassed KNN. SVC outperformed KNN in this case due to a large number of features and limited training data. GaussianNB’s F1 score (60.89%) was relatively low in comparison to the other two statistical models in this experiment. GaussianNB assumed that all features were independent, which is rarely the case in reality. This constrained the algorithm’s application and is the primary cause for the algorithm’s poor F1-scores.

**Figure 15:**
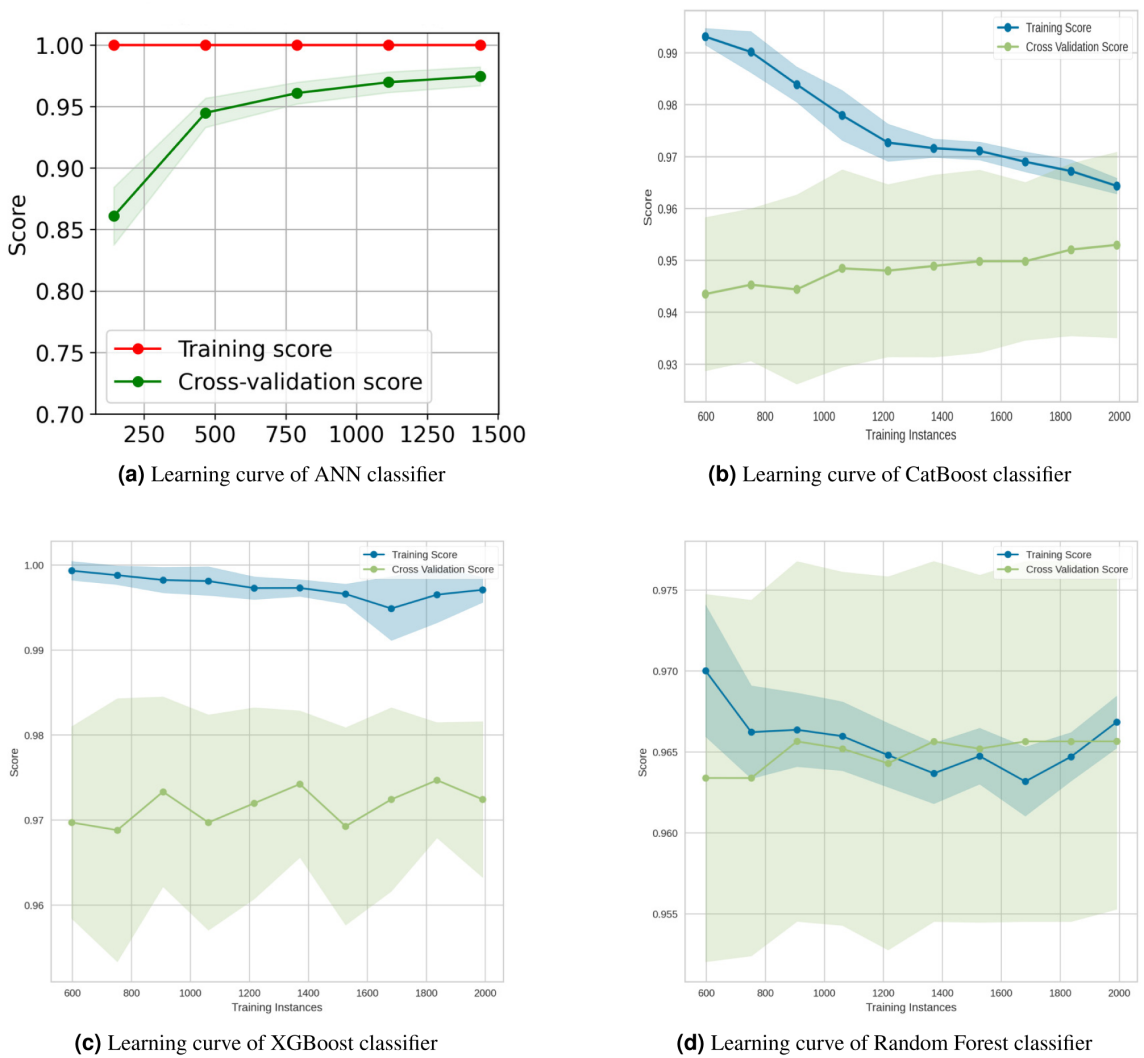
Learning curves for the top four classification algorithms.

**Figure 16:**
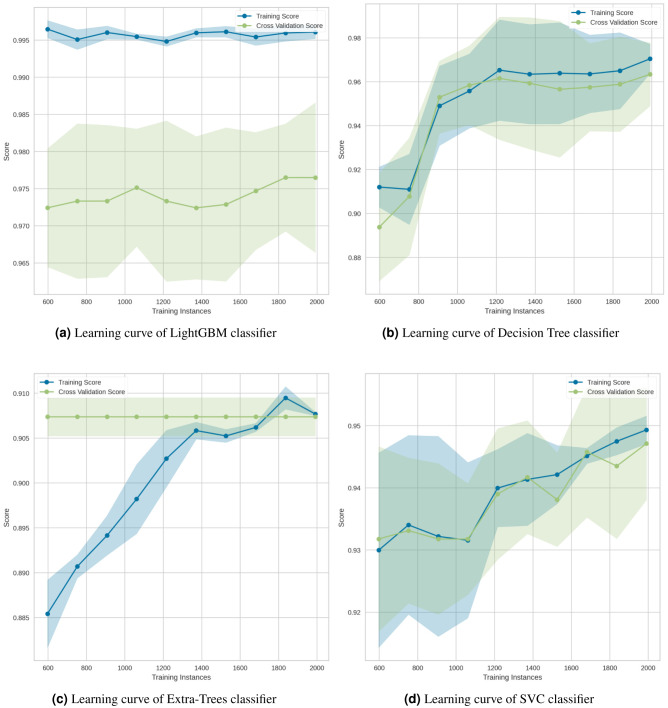
Learning curves for the (A) LightGBM, (B) Decision Tree, (C) Extra-Trees and (D) SVC classification algorithms.

**Figure 17:**
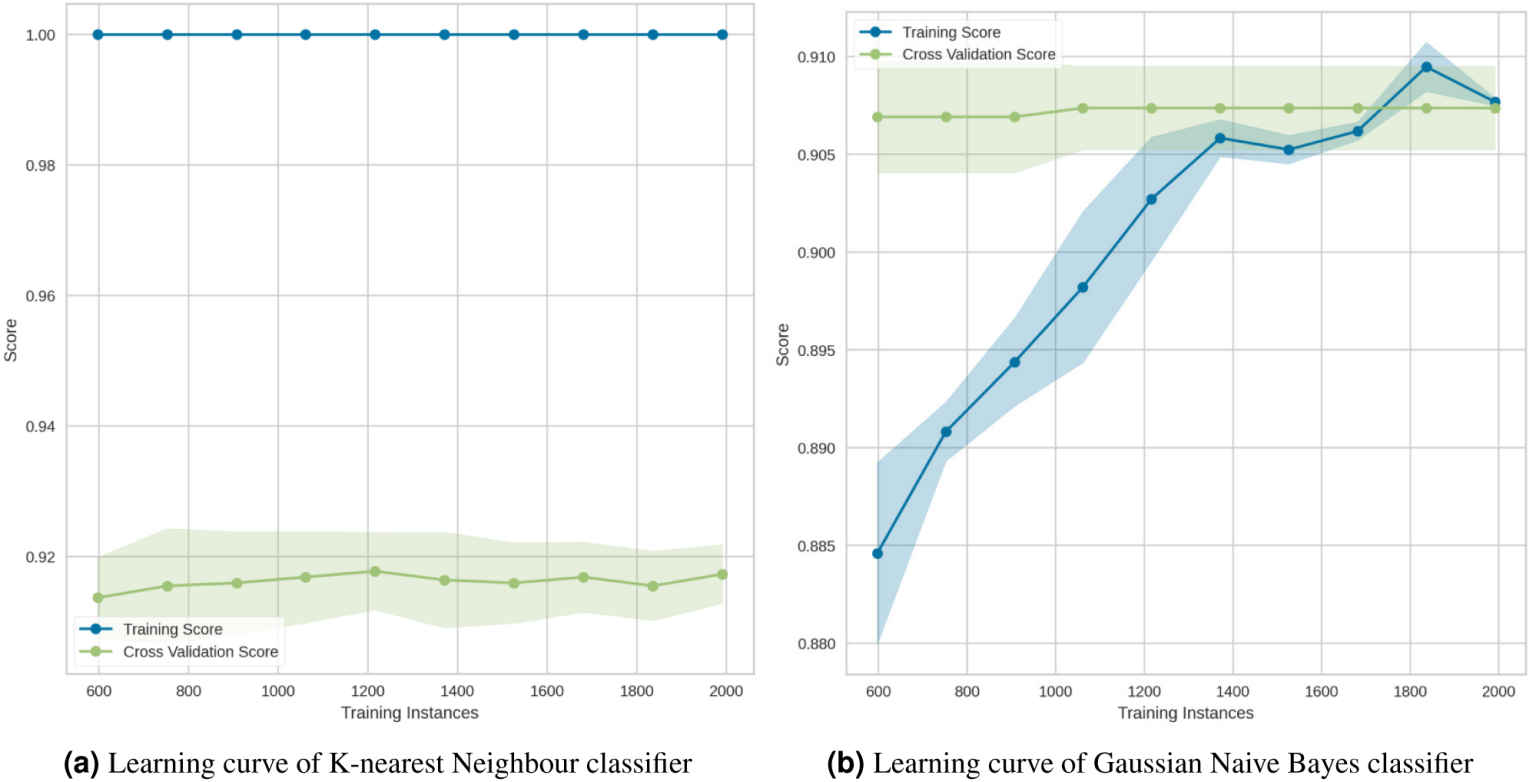
(A–B) Learning curves for the lowest two classification algorithms.

The learning curve is a visualization tool that was used in this experiment to determine the advantage of adding additional training data to our model. It illustrated the connection between the training and test scores for a machine learning model that has a variable number of training samples. Generally, while plotting the learning curve, the cross-validation approach was used. We used the Python Yellowbrick package to visualize the learning curve. In the above graphs Figs. 15–17 the accuracy score for the train set was denoted by the term “Training Score”, whereas the accuracy score for the test set was denoted by the term “Cross-Validation Score”.

The learning curves of the top four classification algorithms are depicted in Fig. 15. The learning curve for the ANN classifier in Fig. 15A showed that the training and test scores have not yet converged, so potentially this model would benefit from adding more training data. Adding more data as row increases diversity. This reduces the generalization error, since training the model with more examples makes it more general. The Akaike information criterion and the Bayesian information criterion (Stoica & Selen, 2004) both show that adding more data always improves models, while adding parameter complexity beyond the optimum reduces model quality. From the Catboost classifier Fig. 15B, it has been seen that adding more training instances would increase the generalization. The XGBoost classifier (Fig. 15C) would benefit from adding more training data as it was suffering from high variance. Finally, the training score of the Random Forest Classifier (Fig. 15D) revealed the degree of underfitting as it falls, while the cross-validation score (green line) fluctuated.

The learning curve for the LightGBM classifier in Fig. 16A showed that there is a large gap between the errors, which indicates that it needs more data to improve. As illustrated in the Decision Tree classifier (Fig. 16B), increasing the number of training cases improved generalization. The Extra-Trees classifier (Fig. 16C) indicated that, regardless of the amount of data fed into the model, the model is incapable of representing the underlying relationship and has a high level of systematic error. Lastly, the SVC classifier (Fig. 16D) demonstrated that the learning curves for testing and training occasionally converged on similar values.

The learning curve for the KNN in Fig. 17A showed that, regardless of the number of training samples, the training score (red line) was maximized. This demonstrated significant overfitting. Finally, the Gaussian Naive Bayes (Fig. 17B) suggested that the model has a low degree of generalization and that it could be simplified by using fewer complex features.

The experiment and interpretation of the results indicate that neural networking classifiers outperform tree-based and statistical-based classifiers on this dataset. As a result, it is recommended to consider a neural network classifier when using this dataset to predict thyroid risk.

## Conclusion

This study presents an experimental study for different machine learning algorithms: neural networking classifiers, tree-based classifiers, statistical classifiers to predict thyroid risk. These algorithms are employed on a particular dataset, namely Sick-euthyroid, to predict the thyroid risk. For evaluating the employed algorithms, precision, recall, F1 score, and accuracy are calculated. Among all the algorithms, the ANN classifier outperforms others with an accuracy of 0.9587. The CatBoost and XGBoost classifiers come second and third with the accuracy of 0.9538 and 0.9533, respectively. The extensive experiments and analyses depict that the neural networking classification algorithms provide better results in terms of accuracy and F1 measure on the Sick-euthyroid dataset to predict the thyroid risk. Therefore, it is recommended to consider neural networking classifiers over other machine learning techniques while using this Sick-euthyroid dataset for thyroid risk prediction.

## Additional Information and Declarations

### Data Availability

The following information was supplied regarding data availability: The code is available at GitHub: https://github.com/saimasharleen/thyroid-dataset. The dataset is available at the UCI Machine Learning Repository: https://archive.ics.uci.edu/ml/datasets/thyroid+disease.
